# Defect-implantation for the all-electrical detection of non-collinear spin-textures

**DOI:** 10.1038/s41467-020-15379-6

**Published:** 2020-03-30

**Authors:** Imara Lima Fernandes, Mohammed Bouhassoune, Samir Lounis

**Affiliations:** Peter Grünberg Institut and Institute for Advanced Simulation, Forschungszentrum Jülich and JARA, D-52425 Jülich, Germany

**Keywords:** Magnetic properties and materials, Surfaces, interfaces and thin films

## Abstract

The viability of past, current and future devices for information technology hinges on their sensitivity to the presence of impurities. The latter can reshape extrinsic Hall effects or the efficiency of magnetoresistance effects, essential for spintronics, and lead to resistivity anomalies, the so-called Kondo effect. Here, we demonstrate that atomic defects enable highly efficient all-electrical detection of spin-swirling textures, in particular magnetic skyrmions, which are promising bit candidates in future spintronics devices. The concomitant impurity-driven alteration of the electronic structure and magnetic non-collinearity gives rise to a new spin-mixing magnetoresistance (XMR_defect_). Taking advantage of the impurities-induced amplification of the bare transport signal, which depends on their chemical nature, a defect-enhanced XMR (DXMR) is proposed. Both XMR modes are systematised for 3*d* and 4*d* transition metal defects implanted at the vicinity of skyrmions generated in PdFe bilayer deposited on Ir(111). The ineluctability of impurities in devices promotes the implementation of defect-enabled XMR modes in reading architectures with immediate implications in magnetic storage technologies.

## Abstract (web summary)

Precise detection of magnetic skyrmions is a key prerequisite to exploit them in future magnetic storage technologies. The authors, using first principles studies, propose two novel spin-mixing magnetoresistance effects enabled by defects, which allow for a highly efficient all-electrical detection of spin-swirling textures.

## Introduction

Defects are inherent to all devices and materials. Being unavoidable, they dramatically reshape transport properties, often negatively, and thus are key ingredients in settling the competitiveness of newly proposed technologies and therefore their survival. In the context of spintronics^1^ exploiting the spin rather than the charge to carry information, defects can reduce the efficiency of magnetoresistance effects^2,3^ in current perpendicular-to-plane geometries such as the giant magnetoresistance (GMR)^4,5^ or tunneling magnetoresistance (TMR)^6,7^. Impurities intrinsically can alter the conductance by increasing it or reducing it, as shown for various constrictions^8–11^, giving rise to inelastic transport channels allowing the exploration of electron-bosons interactions^12–14^ while generating extrinsic contributions to Hall effects^15–20^ in current-in-plane geometries. All of this is not surprising since the very fundamental Kondo effect^21^ results from diluted magnetic impurities leading to an anomalous behavior of the resistance at low temperature^22^.

Recently a new kind of magnetoresistance effect, the spin-mixing magnetoresistance (XMR), has been discovered^23,24^, which in contrast to the GMR or TMR effect enables the all-electrical detection of non-collinear magnetic states, such as magnetic skyrmions^25,26^ and spin spirals, with a non-magnetic electrode. Thus, XMR provides an appealing detection tool of importance in establishing spin-swirling textures with chiral or topological protection properties, in particular skyrmions, as future bits for information technology^27^.

While several investigations were devoted to the impact of inhomogeneities on the motion and stability of skyrmions^28–39^, their implications in their electrical detection are yet to be determined. Similarly to the GMR and TMR effects, it is often expected that defects would reduce the XMR efficiency.

**Fig. 1:**
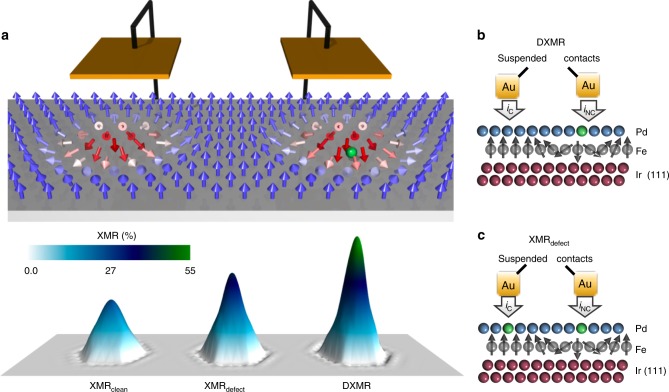
All-electrical detection of magnetic skyrmions in the presence of defects. **a** Illustration of a tunneling transport experiment to read magnetic skymions on fcc-PdFe bilayer on the Ir(111) surface away from (left) or at the vicinity of (right) a single-atomic defect indicated by a green sphere. The spin-mixing of the electronic structure within the non-collinear environment leads to changes on the tunneling conductance allowing the magnetic data information to be sensed in a current perpendicular-to-plane geometry. The various XMRs signals, for the clean system and in the presence of defects, are shown on the lower panel. Depending on the reference background two types of XMR signals can be measured, **b** while the clean ferromagnetic substrate leads to the defect-enhanced XMR (DXMR), **c** the substrate-defect complex background leads to the XMR_defect_.

In this article, we demonstrate from a full ab initio approach that contrary to the current wisdom atomic defects are of technological importance in reading non-collinear spin-states since they enable a highly efficient spin-mixing magnetoresistance signal, where the reference transport signal is amplified by many of the investigated impurities (see Fig. 1a). We envision scenarios, where impurities are manipulated atom-by-atom^40^, implanted^41^ or spontaneously generated via intermixing mechanisms. We introduce defect-enabled XMR modes in order to evaluate the potential of defects in magnifying simultaneously the transport and XMR signals. We perform systematic simulations of atomic resolved transport measurements as probed within scanning tunneling microscopy/spectroscopy (STM/STS) of 3*d* (V, Cr, Mn, Fe, Co, Ni) and 4*d* (Nb, Mo, Tc, Ru, Rh) transition metal defects implanted in the Pd surface layer covering the fcc-Fe monolayer deposited on Ir(111) surface. The latter substrate is known to host few nanometers-wide magnetic skyrmions^42–47^ stabilized by the presence of Dzyaloshinskii-Moriya interaction^48,49^. We identified the different mechanisms conspiring to make the universal trends of the various XMRs, as function of the impurities electronic states, distinct from each other. We put forward the defect-enabled XMR effects as key-reading tools in man-engineered defective substrates with immediate implications for device applications in the context of non-collinear states.

## Results

### Impact of defects on the all-electrical detection

The all-electrical detection of single magnetic skyrmions via the XMR effect^23,24,50^ is made possible via the non-collinearity of the magnetic moments of the Fe layer and the presence of spin-orbit interaction, which lead to a spin-mixing of the electronic states living initially in the majority- and minority-spin channels. Within tunneling transport experiments, these mixed states are detectable with a non-magnetic electrode (Fig. 1a). In the case of PdFe/Ir(111) substrate, the XMR effect as probed in STM/STS is particularly enhanced for resonant interface and surface states^51^, which result from various hybridization mechanisms being affected by relativistic effects.

We consider a skyrmion passing at the vicinity of a defect as illustrated schematically in Fig. 1. Here, it is convenient to define two types of XMR signals contingent on the reference measurement with respect to which the efficiency of the MR effect is calculated. The reference point can be either the clean (defect-free) (see Fig. 1b) or the substrate-defect complex (see Fig. 1c), both in the ferromagnetic configuration. The former leads to the defect-enhanced XMR (DXMR) while the latter is a XMR signal obtained in the presence of the defect and denoted in the following XMR_defect_. In both cases, we calculate the percent deviation of the conductance on top of a skyrmion from that of a reference point within a collinear magnetic region. In a device, such reference points have to be identified, for instance, after saturating the magnetic state by applying a magnetic field. Then the regions with and without defects can be prospected and identified through the change in the transport signal. We note that the XMR signal collected from an STM experiment on a pinned skyrmion^24^ naturally includes the contribution of the pinning defect and should thus correspond to what we name DXMR.

Based on the Tersoff-Hammann approach^52^, the differential conductance $\frac{\mathrm{d}I}{\mathrm{d}V}$ at a given bias voltage *V*_bias_ measured within STM/STS, equipped with a non-magnetic tip, is proportional to the local density of states (LDOS) of the substrate at the energy *E* = *E*_F_ + *eV*_bias_ obtained in vacuum at the tip position. While our focus is the differential conductance, the definitions given below could be extended to the constant-current mode of STM (see Supplementary Note 1). Here, we consider the tip located in second vacuum layer above the Pd substrate as indicated in Fig. 1b, c, thus the efficiency of these magnetoresistance effects are calculated from:

1 $${\text{XMR}}_{\text{defect}}(E)=\frac{{\,\text{LDOS}}_{\text{NC}}^{\text{defect}\,}(E)-{\,\text{LDOS}}_{\text{C}}^{\text{defect}\,}(E)}{{\,\text{LDOS}}_{\text{C}}^{\text{defect}\,}(E)},$$

which is analogue to the conventional defect-free XMR,

2 $${\text{XMR}}_{\text{clean}}(E)=\frac{{\,\text{LDOS}}_{\text{NC}}^{\text{clean}\,}(E)-{\,\text{LDOS}}_{\text{C}}^{\text{clean}\,}(E)}{{\,\text{LDOS}}_{\text{C}}^{\text{clean}\,}(E)},$$

where all LDOS are obtained in the clean region. C and NC correspond respectively to collinear and non-collinear magnetic regions, as shown in Fig. 1b, c. It is important to note that XMR_defect_ and XMR_clean_ effects are fundamentally different since the former is settled by the defect’s electronic states and how they react to the presence of a non-collinear spin-texture.

The proposed DXMR efficiency is extracted from:

3 $${\text{DXMR}}\,(E) = \frac{{\,\text{LDOS}}_{\text{NC}}^{\text{defect}\,}(E)-{\,\text{LDOS}}_{\text{C}}^{\text{clean}\,}(E)}{{\,\text{LDOS}}_{\text{C}}^{\text{clean}\,}(E)}\\ = {\text{XMR}}_{\text{clean}}(E)+\frac{{\,\text{LDOS}}_{\text{NC}}^{\text{defect}\,}(E)-{\,\text{LDOS}}_{\text{NC}}^{\text{clean}\,}(E)}{{\,\text{LDOS}}_{\text{C}}^{\text{clean}\,}(E)},$$

which obviously measures the enhancement of the XMR efficiency by the defect with respect to the signal obtained in the defect-free region. The latter equation shows that the DXMR signal is related to the “traditional” defect-free XMR effect and it is obviously shaped by how the tunneling matrix elements involving the defect are different from those of the defect-free substrate. In fact, by re-expressing it as:

4 $$1+\,{\text{DXMR}}\,(E) =	\, \left(1+{\text{XMR}}_{\text{defect}}(E)\right)\cdot \left(1+{D}_{\text{C}}(E)\right)\\ =	\, \left(1+{\text{XMR}}_{\text{clean}}(E)\right)\cdot \left(1+{D}_{\text{NC}}(E)\right),$$

where

5 $${D}_{\text{NC}}(E)=\frac{{\,\text{LDOS}}_{\text{NC}}^{\text{defect}\,}(E)-{\,\text{LDOS}}_{\text{NC}}^{\text{clean}\,}(E)}{{\,\text{LDOS}}_{\text{NC}}^{\text{clean}\,}(E)}$$

and

6 $${D}_{\text{C}}(E)=\frac{{\,\text{LDOS}}_{\text{C}}^{\text{defect}\,}(E)-{\,\text{LDOS}}_{\text{C}}^{\text{clean}\,}(E)}{{\,\text{LDOS}}_{\text{C}}^{\text{clean}\,}(E)}$$

one deduces that the DXMR signal can be interpreted as the XMR_defect_ signal altered by the bare signal provided by the defect. The larger the latter is, the better the detection of the magnetic skyrmion. We note that *D*_C_ can be obtained in the saturared magnetic state prior to the generation or injection of the non-collinear spin-textures.

**Fig. 2:**
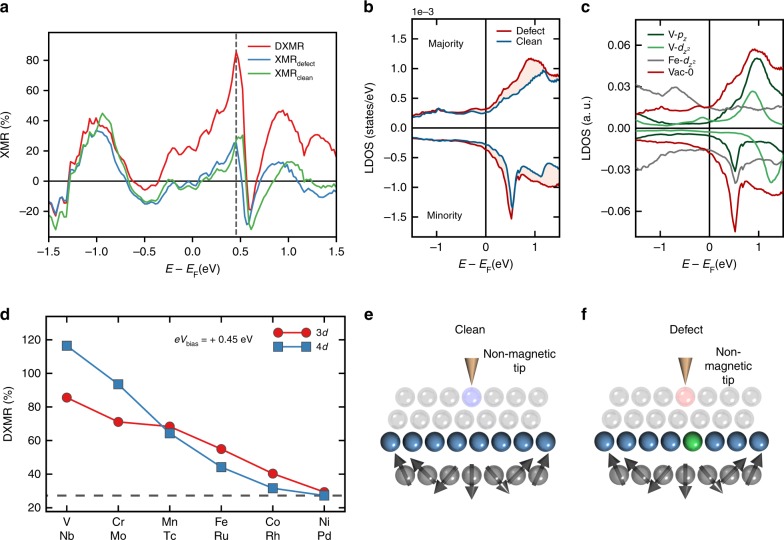
Efficiency of the XMR signal and the electronic structure. **a** Comparison of various energy-resolved XMR signals measured on top of the skyrmion's core with V impurity located close to skyrmion's core. The dashed line indicates the energy at which the DXMR reaches the maximum efficiency. **b** Electronic structure in the vacuum on top of the skyrmion's core resolved into majority and minority spin-channels for the clean system (blue line) and with a V-impurity (red line). **c** Superposition of the electronic states in vacuum of V and Fe at the skyrmion's core. Note that arbitrary units are used to include all of the curves in the same plot. **d** Impact of chemical nature of the defect on the DXMR signal at an injection energy *eV*_bias_ = 0.45 eV for 3*d* (red circles) and 4*d* (blue squares) impurities located close to the skyrmion center. The gray dashed line indicates the signal of the XMR_clean_. **e**, **f** Illustrative legend for the STM-tip probing the clean (defective) substrate at the location defined by the light blue (red) sphere, whose color corresponds to the LDOS plotted in (**b**). Vacuum is simulated by empty cells, schematically represented as spheres.

For conciseness, we focus our first analyses on the impact of a V impurity at the closest vicinity of the skyrmion’s core on the various XMRs, whose energy-resolved signals are plotted in Fig. 2a. The investigated skyrmion has a diameter *D*_Sk_ ≈ 2.2 nm and we recall that in the skyrmion’s core, the magnetic moment of the substrate Fe atom is flipped with respect to the ferromagnetic surrounding. Among the XMR signals, the DXMR efficiency is the largest with an impressive ≈85%, i.e., an increase of ≈230% with respect to the defect-free signal, for an energy injection *eV*_bias_ = +0.45 eV (Fig. 2a, red curve). As deduced from Eq. (4), this large efficiency is just an indication that the defect enhances the bare tunneling transport signal. For comparison, the XMR_clean_ (Fig. 2a, green curve) and XMR_defect_ (Fig. 2a, blue curve) signals reach a similar efficiency around 26%. In the whole investigated energy window, the latter two signals have a shape, which in general, seems similar to the DXMR signal. Moreover, we note that the large efficiencies observed in the various XMR effects obtained on the basis of the differential conductance measurements are present in the constant-current mode (see Supplementary Note 1).

The XMR_clean_ efficiency is shaped by the non-collinearity of the substrate magnetic moments and the presence of spin-orbit interaction. Two additional mechanisms, enabled by the presence of the defect, contribute to the XMR_defect_: (i) impurity-induced modification of the substrate’s non-collinearity and (ii) hybridization of the electronic states of the defect with the substrate Fe atoms and the way these states decay into vacuum, which settles the tunneling matrix elements. In contrast, the DXMR signal is more complex since it involves mechanisms active for XMR_clean_ and XMR_defect_ weighted by the enhancement factors introduced by the defect. The latter would be useful to enhance weak transport signals that can be of importance for buried skyrmions located away from the probing electrodes.

### Enhancement of the bare signal and of the DXMR efficiency

The reference background for both the XMR_clean_ and the DXMR signal is the defect-free region away from the skyrmion (collinear magnetic region). Thus, to understand the enhancement of the DXMR effect, we invoke Eq. (4) and analyse the vacuum LDOS just above the skyrmion core with and without the implanted V-atom (Fig. 2e, f). As illustrated in Fig. 2b, the vacuum electronic states of both spin-channels are strongly amplified by the presence of the V-defect affecting the differential conductance by virtue of the Tersoff-Hamann approximation (see Supplementary Note 2 for details related to the electronic structure of the collinear magnetic region). Here one identifies the main advantage of a substrate hosting impurities. The overall STM/STS signal is increased, which greatly facilitates the detection of buried skyrmions, as it is for PdFe/Ir(111) surface. Thus, defects can act as mediating probes for the electrical detection. The observed vacuum states originate from the decay of the electronic states of the V-defect and Fe layer plotted in Fig. 2c, as can be noticed from the one-to-one correspondence between the atomic virtual bound states and the features probed in vacuum. The orbital-resolved LDOS, e.g., the *p*_z_ and ${d}_{{\text{z}}^{2}}$ states, leading to the largest tunneling matrix elements are shown in particular for the V defect and for the Fe atom at the skyrmion’s core. The latter defines the spin-frame of reference in which the LDOS is plotted and the convention to denote the majority- and minority-spin states.

Because of the large exchange splitting of V, the hybridization is weak between the occupied majority-spin states of Fe and the unoccupied ones of V, giving rise to the ${d}_{{\text{z}}^{2}}$ and *p*_z_ virtual bound states located at ≈+0.9 eV and ≈+1.0 eV, respectively (see upper channel of Fig. 2c). Recalling that V replaces a Pd atom from the defect-free substrate, we expect thus a larger intensity of the features observed in vacuum because of the additional impurity-states. Similar conclusions can be drawn for the opposite spin channel. Interestingly, an unoccupied *p*_z_ virtual bound state at  ≈+0.53 eV is induced by the Fe minority-spin state in the LDOS of V (see lower channel of Fig. 2c), with a larger intensity than the one obtained in the clean Pd-overlayer.

### Systematic trends

The enhancement of bare transport signal detected within the DXMR signal with respect to the conventional XMR is not limited to the V impurity but occurs for all investigated implanted defects of the 3*d* and 4*d* series. The factor of enhancement depends, however, on the chemical nature of the impurities as well as on the injection energy. In Fig. 2d, we systematically collect the efficiency of the DXMR as function of the impurities atomic number for an injection energy of *eV*_bias_ = +0.45 eV. Overall, the DXMR ratio decreases when increasing the atomic number of the defects. For the 3*d* elements, V leads to the highest efficiency, which is larger than the 30% efficiency induced by Ni. The latter defect does not alter the defect-free XMR efficiency. Interestingly, defects from the beginning of the 4*d* series lead to an impressive enhancement of the signal, reaching almost 116% for Nb followed by 94% for Mo which translate to a increase of about 350% and 260%, respectively, with respect to the defect-free XMR efficiency. This implies that the best impurities for the enhancement of the bare tunneling transport signal are early transition elements with a preference for the 4*d* series.

**Fig. 3:**
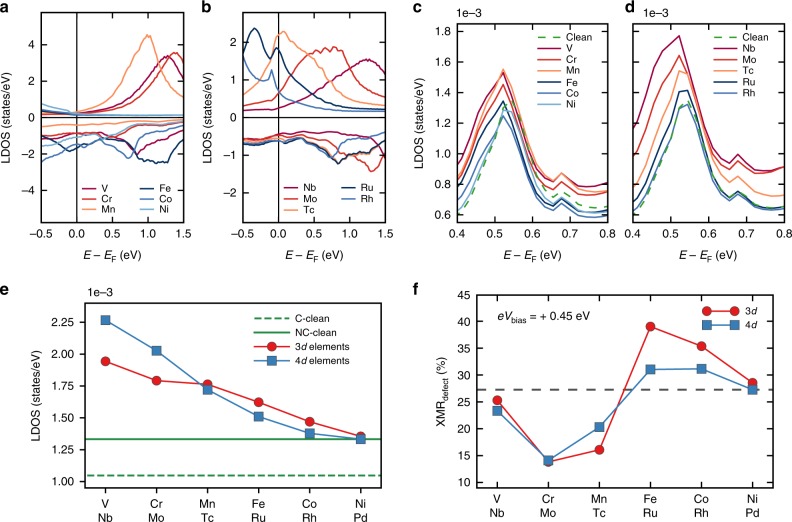
Impact of defects on the electronic structure and on the XMR_defect_ signal. Local density of states (LDOS) of the defect within the collinear configuration for **a** the 3*d*-inatom series **b** the 4*d*-inatom series. The minority-spin channels of the LDOS of the vacuum on top of the core of the skyrmion with a **c** 3*d* and **d** 4*d* impurity close to the skyrmion core. **e** Chemical trend of the total LDOS of the vacuum on top of the core of the skyrmion at +0.45 eV for the 3*d*-elements (red line) 4*d*-elements (blue line), where the total LDOS is the sum of the LDOS of the minority- and majority-spin states. The green full line (dashed line) indicates the LDOS of the vacuum within the non-collinear (collinear) magnetic region for the defect free-system substrate at +0.45 eV. **f** Impact of band-filling on the defect spin-mixing magnetoresistance (XMR_defect_) at an injection energy *eV*_bias_ = 0.45 eV. The gray dashed line indicates the signal of the XMR_clean_.

The behavior of the DXMR signal as function of the chemical nature of the defect can be directly related to the filling of the impurities electronic states. By moving from left to right across the 3*d* (4*d*) atomic row of the periodic table the unoccupied states of the impurities shift towards the Fermi energy becoming partially or almost fully occupied at the end of the series as can be seen in Fig. 3a, b for the collinear magnetic states. Interestingly, this sequence is accompanied with a transition from an antiferromagnetic coupling to the substrate for V (Nb), Cr (Mo), and Mn (Tc) to a ferromagnetic coupling for Fe (Ru), Co (Rh), and Ni (Pd). The decrease of the impurities LDOS upon the filling of the electronic states, as illustrated by Fig. 3e, explains the DXMR trend plotted in Fig. 2d as well as the very large ratio induced by the 4*d* impurities when compared to the 3*d* ones. The smaller exchange splitting of the 4*d* elements increases the possibility of having large LDOS around the Fermi energy.

Paying closer attention to the electronic features of the vacuum on top of the core of the skyrmion responsible for the discussed effect for the 3*d* and 4*d* series (see Fig. 3c, d), one identifies that the peaks obtained for the antiferromagnetic V (Nb), Cr (Mo) and Mn (Tc) are more intense than those of the ferromagnetic Fe (Ru), Co (Rh) and Ni (Pd). Here, two concomitant mechanisms are at play. First the hybridization strength is known to decrease from left-to-right and to increase from up-to-down across the transition element series of the periodic table^37^. Second, the *p*_z_ state induced in the electronic structure of the impurities depends on the magnetic coupling because of the switch of the spin-nature of the impurity states hybridizing with the minority-spin states of Fe. Thus, more impurity states are available when the spin-alignment is rather ferromagnetic.

Contrasting the DXMR behavior, the XMR_defect_ for an injection energy *eV*_bias_ = +0.45 eV when plotted as function of the atomic number (see Fig. 3f) exhibits a S-like shape with a maximum and a minimum close to the middle of the series with the efficiency increasing up to about 56% with respect to the defect-free signal. At this particular injection energy, the XMR_defect_ signal is better magnified by impurities with more than half-filled *d*-states instead of the early transition elements, which are better suited to enhance the bare transport signal as previously discussed.

**Fig. 4:**
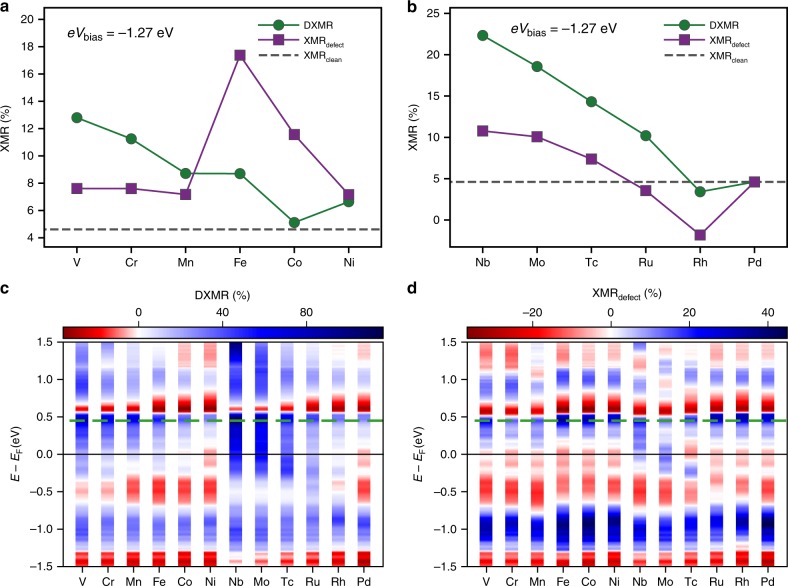
Impact of the bias voltage on the defect-enabled XMR modes. Dependence of the DXMR signal (green circle) and XMR_defect_, (purple squares), on the atomic number of the **a** 3*d* and **b** 4*d* impurities at an injection energy *eV*_bias_ = −1.27 eV. The gray dashed line indicates the signal of the XMR_clean_. Bias voltage dependence of the **c** DXMR and **d** XMR_defect_ signal for 3*d* and 4*d* defects. The color bars indicate the magnitude of the signal and the dashed green line shows the injection energy *eV*_bias_ = 0.45 eV studied in Figs. 2 and 3.

Figure 4a, b shows a comparison of the XMR_defect_ and DXMR efficiencies at the injection energy *eV*_bias_ of −1.27 eV for the 3*d* and 4*d* defects. One notices that by changing the injection energy, the S-shape observed for XMR_defect_ at 0.45 eV can be reversed and the location of the extrema can be strongly shifted. The usual XMR_clean_ efficiency is found rather low with a value of 4.6% but thanks to the defects, the XMR efficiencies increase. Among the 3*d* impurities, Fe and Co induce XMR_defect_ efficiencies of respectively 17.4% and 11.5%, i.e., an increase of respectively 277% and 151% with respect to XMR_clean_, which are a factor two larger than the DXMR ones of 8.7% and 5.1%, respectively.

In general, we note that the XMR_defect_ signal for the 3*d* elements keeps the S-shape for most of the bias voltages, while the pattern obtained for the 4*d* elements can change. An overview on the simultaneous dependence of the defects-enabled XMR efficiencies on the atomic number of the impurities and the magnitude of the bias voltage is presented in Fig. 4c, d. The signal corresponding to XMR_clean_ is the one of Pd and the dashed line indicates the particular case of *eV*_bias_ = 0.45 eV. One notices that the pattern of intensities can be manipulated to a great extent by changing the nature of the defects. There is an effective dispersion of the blue and red “band” having distinct bandwidths depending on the energy or impurities atomic number. Also, one sees how the white “bands”, i.e., those corresponding to a weak XMR signal, are modified. Early transition elements seem to generate most of the widening of the blue bands, i.e. large DXMR efficiencies with a positive sign. Interestingly white bands emerge within the large blue bands characterizing the 4*d* elements. A large difference between the patterns obtained for positive and negative bias voltages is observed for XMR_defect_, with more blue-red sequences observed at positive energies. This is probably due to the minority-spin states of the substrate that are more sensitive to the hybridization with the impurity states.

### Real-space XMRs contrast and inhomogeneous non-collinearity

**Fig. 5:**
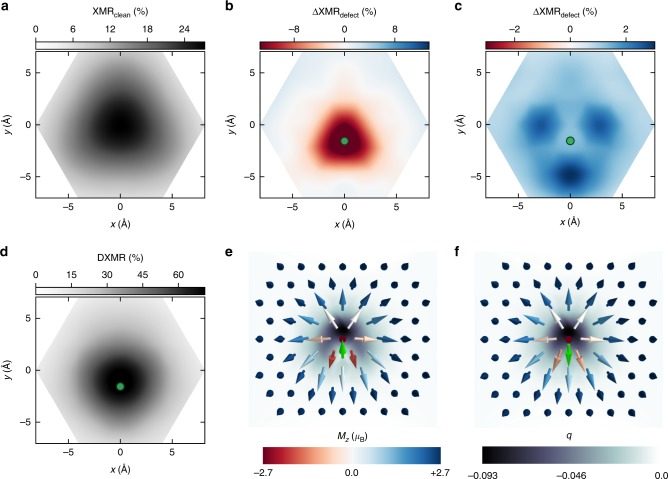
Impact of single-atomic defects on XMR-signals of skyrmions. **a** Defect-free XMR signal compared to **d** DXMR signal induced by a Cr impurity. Difference between XMR_defect_ and XMR_clean_ plotted for **b** Cr and **c** Ni impurities close to the core of skyrmion. The green circle indicates the position of the impurity. The corresponding skyrmion magnetic textures and the topological charge monitoring the non-collinearity are shown in (**e**) for Cr and (**f**) for Ni, where the green arrow represents the impurity magnetic moment while the rest of arrows are those of the Fe atoms. The red-blue color bar represents the magnitude of the out-of-plane magnetization (*M*_z_) of each Fe atom while the grayscale color bar is used for the topological charge computed for triangles defined by sets of three nearest neighboring atoms. An interpolation is then performed between the discrete values of the charge.

The XMR efficiency can be of use to detect slight changes in the magnetic texture. The XMR_clean_ and XMR_defect_ depend on the opening angle between neighboring magnetic moments. In the defect-free region, the measurable electrical contrast is expected to exhibit a highly symmetric shape, translating the symmetry of the magnetic texture, with the highest intensity at the center of the skyrmion (see Fig. 5a). At the vicinity of a defect, the skyrmion experiences an asymmetric environment impacting its spin-texture and consequently the all-electrical XMR contrast. This is better grasped by visualizing the difference ΔXMR_defect_ = XMR_defect_ − XMR_clean_ as done in Fig. 5b, c for Cr and Ni impurities. For Cr, the XMR_defect_ signal strongly decreases at the close vicinity of the impurity since the strong exchange coupling of the impurity and the substrate atoms reduces the surrounding non-collinearity (see Supplementary Note 3 and Fig. 5e), which lowers the XMR ratio. For Ni, however, the change in the XMR signal (Fig. 5c) is off-set from the defect with the pattern being less intense than for Cr, which corresponds to the modifications induced in the magnetic texture shown in Fig. 5f. We note that in Fig. 5e, f the non-collinearity is probed in terms of the topological charge density (more details are given in Supplementary Note 4). For the investigated bias voltage of 0.45 eV, the DXMR signal tend to be not that sensitive to the impurity-induced non-collinear modifications and reaches a maximum value on top of all defects. As an example, the Cr-related DXMR signal at the same bias energy is illustrated in Fig. 5d when the impurity is close to the core of the skyrmion.

## Discussion

In this work, we explore the possibility of using defects to detect non-collinear magnetic textures using all-electrical means. By strongly modifying the tunneling matrix elements, defects can increase the intensity of the background transport signal. This would prove useful in case of buried non-collinear spin-textures, which are difficult to access with surface probe techniques. To track the impact of impurities, we defined the XMR_defect_ and DXMR, which monitor the enhancement of the XMR efficiency induced by the presence of the defects. DXMR can even benefit from the amplification provided by the modification of the bare transport signal. Since both proposed defect-enabled XMR modes could be simultaneously accessed, it is expected that one can select the one with the best efficiency while performing the measurements.

From the obtained general trends upon filling of the impurities electronic states, our investigations at the bias voltage of *eV*_bias_ = +0.45 eV point to the use of early transition elements embedded in Pd/Fe/Ir(111) to enhance the bare tunneling transport patterns, while elements with more than half-filled *d*-shells seem to be better suited for a better detection via XMR_defect_. Besides the atomic number of the impurities, the applied bias voltage is an important parameter for the various effects shaping the XMR efficiencies, which can modify the general trends to a great extent.

The Tersoff-Hamann approximation^52^ considered for the evaluation of the differential conductance assumes a tip with an *s*-orbital. Changing the nature of the tip-orbitals modifies the tunneling matrix elements dictating which substrate’s states contribute to transport^53,54^. The bare transport spectra, and therefore the various XMR, are expected to be altered, which opens a large amount of possibilities to tune the efficiencies of the signals. Obviously, initially “silent” substrate’s states can become active and enhance the all-electrical detection abilities by engineering the nature of the probing electrode. This, in principle, can be done by changing either the tip or by deliberately transferring an atom or a molecule to the tip apex^55–58^.

The magnitude of the defect-enabled XMR efficiencies hinges on the location of the impurities on the substrate. For instance, the defects can be located atop, instead of being embedded in the Pd layer as assumed in the current investigation. Our proof-of-concept study has, however, predictive value since the related formation energies (see Supplementary Note 5) indicate that all investigated 4*d* impurities and a couple of 3*d* atoms (V, Cr) have a clear preference to be embedded in the Pd layer, while Mn and Ni prefer to sit atop the substrate.

By providing a path to enhance in various ways the all-electrical detection of non-collinear magnetic textures, harnessing defects and their resulting electronic structure might prove useful for applications. We envision single or a complex defects arranged in a controlled manner, as done by STM or ion-implantation, to enhance in specific regions the electrical detection. Thus, the DXMR and XMR_defect_ techniques can be incorporated in future reading technologies, which enforces the view that controlled engineering of defective-materials is a promising route for device architectures.

## Methods

### Computational details

The electronic structure was determined employing first principles calculations based on density functional theory (DFT) in the local spin density approximation. The calculations were performed with the full-potential scalar-relativistic Korringa-Kohn-Rostoker (KKR) Green function method with spin-orbit coupling included self-consistently^59,60^. The method allows to embed single magnetic skyrmions and defects without the need for periodic supercells. This is performed in a two-steps approach. First, self-consistent calculations of the defect-free and skyrmion-free magnetic slab with periodic boundary conditions are done. Afterwards, the Green function, *G*_0_, of the ferromagnetic substrate is harvested in order to solve the Dyson equation, schematically written as *G* = *G*_0_ − *G*_0_Δ*VG*, in order to obtain the new Green function *G*. Δ*V* describes the change in the potential after embedding the perturbation, which as mentioned earlier consists of a non-collinear spin-texture such as a magnetic skyrmion, an impurity or both. The magnetic textures are obtained in a self-consistent fashion till convergence is achieved.

In practice, the Pd/Fe/Ir(111) slab consists of a fcc-stacked PdFe bilayer deposited on 34 layers of Ir with atomic positions obtained from ab initio^44^. The embedded cluster consists of 124 atoms in total among which 37 Fe atoms (see refs. ^23,37^ and references therein for details). We assume an angular momentum cutoff at *l*_max_ = 3 for the orbital expansion of the Green function and when extracting the LDOS a k-mesh of 200 × 200 is considered.

## Supplementary information

Supplementary Information

## Data Availability

The data that support the findings of this study are available from the corresponding authors on request.

## Code availability

The KKR Green function code that supports the findings of this study is available from the corresponding author on request.
