# Supplementary material for: Defect-implantation for the all-electrical detection of non-collinear spin-textures
Source: Nat Commun. 2020 Mar 30;11:1602. doi: 10.1038/s41467-020-15379-6 (PMC7105493; doi:10.1038/s41467-020-15379-6)
Supplement: Supplementary file 1 — Supplementary Information [file 41467_2020_15379_MOESM1_ESM.pdf]

Supplementary Materials

Defect-implantation for the all-electrical detection of non-collinear spin-textures

Lima Fernandes *et al.*

Supplementary Note 1 - XMR effect and the constant-current mode

In the main manuscript, the results were discussed in terms of the differential conductance ${}^{dI}/{}_{dV}$ accessible via scanning tunneling microscopy. The latter can be utilized in the constant-current mode, leading to a signal that can be theoretically accessed after integration of the local density of states (LDOS) from the Fermi energy up to the bias voltage of interest. The defects-enabled spin-mixing magnetoresistances (XMRs) obtained within the constant-current mode are plotted in Supplementary Figure 1 for the case of V-impurity embedded in the Pd-layer deposited on Fe/Ir(111) surface. Similarly to the Figure 2a of the main text, one sees that the various XMR effects are present and can have a large magnitude with the defect-enhanced XMR (DXMR) signal achieving a maximum of 50% for $eV_{\text{bias}} = +0.53\,\text{eV}$, while the maximum value reached by $\text{XMR}_{\text{defect}}$ is about 10%. Note that the maximum efficiency achieved for the different XMR modes is located at different bias voltages.

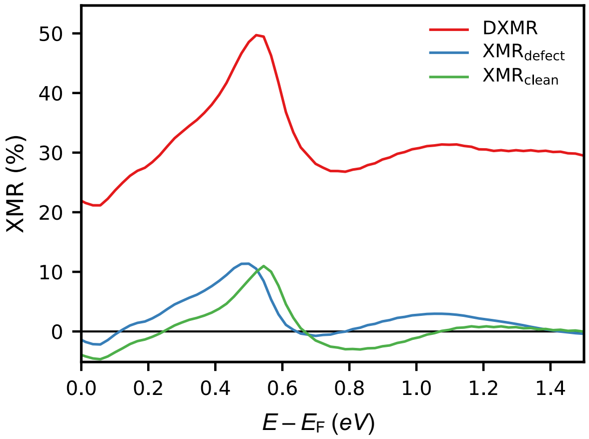

Supplementary Figure 1: XMR effects in the constant-current mode. Various XMR signals obtained within the constant-current mode, i.e. by integrating the LDOS from the Fermi energy E_{F} up to the energy of interest, for a V impurity embedded in the Pd layer deposited on Fe/Ir(111) surface.

Supplementary Note 2 - LDOS in the collinear background

In Supplementary Figure 2, we plot the LDOS of vacuum sites at a distance of 2.2 Å above the PdFe/Ir(111) surface in a collinear state with (red line) and without (blue line) the presence of a V-defect. Similar to the case of the skyrmion shown in the main manuscript Figure 2b, both spin channels are amplified by the presence of the V-defect. Indeed, a large resonance peak is observed in the majority states at $\approx 1.2\,\mathrm{eV}$ in the presence of the defect.

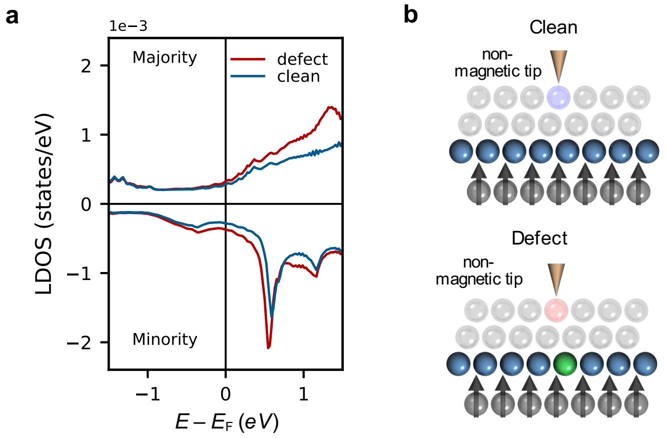

Supplementary Figure 2: Electronic structure in the collinear background. a Electronic structure in the vacuum above the PdFe/Ir(111) surface in a collinear state resolved into majority- and minority-spin channels for the clean system (blue line) and with a V-impurity (red line). **b** Illustrative legend for the STM-tip probing the clean (defective) substrate at the location defined by the light blue (red) sphere, whose color corresponds to the LDOS plotted in **a**. Vacuum is simulated by empty cells, schematically represented as spheres.

Supplementary Note 3 - Magnetic exchange interactions

In Supplementary Table 1, we provide information regarding the impact of the defects on the averaged Fe-Fe nearest-neighbor exchange interaction, $\langle J_{\text{Fe-Fe}} \rangle_{\text{NN}}$, and magnetic interaction between the inatoms and the nearest neighboring substrate atoms, $J_{\text{imp-Fe}}$. These parameters were obtained from our ab-initio calculations utilizing the infinitesimal rotation method [1–3] with the Heisenberg Hamiltonian: $H = -\sum_{ij} J_{ij}\, \mathbf{n}_i \cdot \mathbf{n}_j$, where the unit vectors $\mathbf{n}_i = \mathbf{M}_i/M_i$ define the direction of the atomic magnetic moment $\mathbf{M}_i$ at site i. We note that our mapping leads to the full tensor

of magnetic interactions, which is not discussed in this note. Since the Fe atoms surrounding the defect are not equivalent we averaged the pairwise interactions, which is indicated by the symbol $\langle \quad \rangle$. Throughout this supplementary note, a positive (negative) J describes a ferromagnetic (antiferromagnetic) coupling.

We see that the presence of both the 3*d* and the 4*d* defects reduce locally the exchange interaction among the nearest-neighbor Fe atoms. Atoms from the beginning of the transition elements series couple antiferromagnetically to the Fe substrate atoms in contrast to the elements of the end of series, which prefer a ferromagnetic coupling. For the $3d$ defects the transition from ferromagnetic to antiferromagnetic occurs around Mn, which shows a weak coupling to the Fe-layer. As expected, the magnetic exchange to the Fe substrate atoms is much weaker for $4d$ than for $3d$ defects.

Supplementary Note 4 - Topological charge

We plot in Figure 4e-f of the main manuscript the change of the topological charge of the skyrmion to monitor the modification of the non-collinearity of the spin-texture at the vicinity of defects. We use the discretized version of the topological charge, $\mathcal{Q}$, given as a sum over the solid angle in each triangle obtained by triangulating the entire lattice [4], $\mathcal{Q} = \sum_{\Delta_i} q_i$, with the topological charge assigned to a triangle i:

$$q_i = \frac{2}{4\pi} \arctan\left[\frac{\mathbf{n_k} \cdot (\mathbf{n_l} \times \mathbf{n_m})}{1 + \mathbf{n_k} \cdot \mathbf{n_l} + \mathbf{n_l} \cdot \mathbf{n_m} + \mathbf{n_m} \cdot \mathbf{n_n}}\right], \tag{1}$$

where $\mathbf{n_k}$, $\mathbf{n_l}$ and $\mathbf{n_m}$ are the unit vectors of the three magnetic moments defining the triangle Δ_i.

	3d-elements					
	V	Cr	Mn	Fe	Co	Ni
$J_{\text{imp-Fe}}$	-36.3	-34.7	-6.4	21.5	21.5	13.7
$\langle J_{\text{Fe-Fe}} \rangle_{\text{NN}}$	14.5	14.5	14.3	14.0	15.0	15.6
	4d-elements					
	Nb	Mo	Tc	Ru	Rh	clean
$J_{\text{imp-Fe}}$	-11.4	-15.9	-2.8	5.4	9.7	3.8
$\langle J_{\text{Fe-Fe}} \rangle_{\text{NN}}$	14.6	13.6	13.9	15.2	16.3	16.4

Supplementary Table 1: Magnetic exchange interaction. The isotropic exchange interaction in meV of the defects with the nearest neighboring atoms in the Fe-layer ($J_{\text{imp-Fe}}$) and the isotropic exchange interaction among the nearest neighboring Fe atoms ($\langle J_{\text{Fe-Fe}} \rangle_{\text{NN}}$).

Supplementary Note 5 - Formation energies of 3*d* and the 4*d* implanted defects

In our study, we investigated the XMR signals for impurities embedded in the Pd layers deposited on Fe/Ir(111). Here we explore whether the considered location of the defects would be more preferable than the adatom position by calculating the related formation energy shown in Supplementary Figure 3. This is important to assess the reliability of the predictions made in the main text. A negative energy indicates that the impurity prefers to be embedded in the Pd layer instead of being on top (adatom in the hollow site). We found that all investigated $4d$ atoms as well as some of the $3d$ atoms (V, Cr) prefer to stay in the Pd layer. However, Mn and Ni prefer to sit on top of the surface while Fe and Co do not show a clear preference. So apart from Mn and Ni, considering the atomic defects in the Pd layer is a realistic scenario.

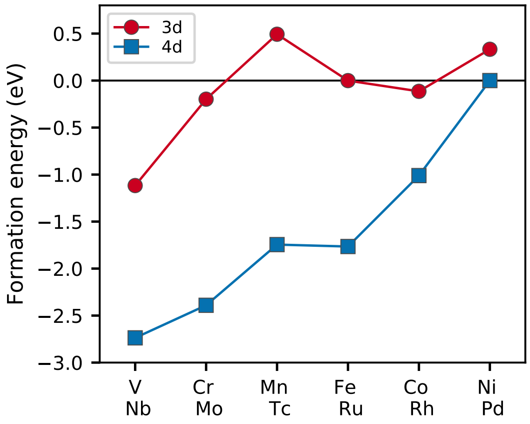

Supplementary Figure 3: Formation energies. Formation energies estimated for the investigated $3d$ and $4d$ impurities. Negative (positive) values indicate the preference to be embedded within (atop) the Pd layer deposited on Fe/Ir(111) surface.

Supplementary References

1. Ebert, H. & Mankovsky, S. Anisotropic exchange coupling in diluted magnetic semiconductors: *Ab initio* spin-density functional theory. *Phys. Rev. B* **79**, 045209 (2009).

2. Liechtenstein, A., Katsnelson, M., Antropov, V. & Gubanov, V. Local spin density functional approach to the theory of exchange interactions in ferromagnetic metals and alloys. *Journal of Magnetism and Magnetic Materials* **67**, 65 – 74 (1987).

3. Lounis, S. & Dederichs, P. Mapping the magnetic exchange interactions from first principles: Anisotropy anomaly and application to Fe, Ni, and Co. *Phys. Rev. B* **82**, 180404 (2010).

4. Yin, G. *et al.* Topological charge analysis of ultrafast single skyrmion creation. *Phys. Rev. B* **93**, 174403 (2016).
